# Vaccine Can Induce CD4-Mediated Responses to Homocitrullinated Peptides *via* Multiple HLA-Types and Confer Anti-Tumor Immunity

**DOI:** 10.3389/fimmu.2022.873947

**Published:** 2022-04-08

**Authors:** Katherine Cook, Wei Xue, Suha Atabani, Peter Symonds, Abdullah Al Omari, Ian Daniels, Sabaria Shah, Ruhul Hasan Choudhury, Daisy Weston, Rachael Metheringham, Victoria Brentville, Lindy Durrant

**Affiliations:** ^1^ Scancell Limited, Biodiscovery Institute, University of Nottingham, Nottingham, United Kingdom; ^2^ The Cancer Vaccine Group, Biodiscovery Institute, University of Nottingham, Nottingham, United Kingdom

**Keywords:** carbamylation, cancer, MHC-II, post-translation modifications, Vimentin

## Abstract

Homocitrullination is the post translation modification (PTM) of the amino acid lysine to homocitrulline also referred to as carbamylation. This PTM has mainly been studied in relation to autoimmune diseases including rheumatoid arthritis. Homocitrullination of lysines alters their charge which can lead to generation of neoepitopes that are differentially presented by MHC-II and induce modification-specific immune responses. Homocitrullination is often considered a process which triggers autoimmune disease by bypassing self-tolerance however, we suggest that homocitrullination may also have an alternative role in immune responses including protection against cancer. Here we demonstrate that immune responses to homocitrullinated peptides from three different proteins can be induced *via* multiple HLA-types. Immunization of Balb/c or HLA-transgenic DR4 and DR1 mice can induce modification-specific CD4 mediated IFNγ responses. Healthy human donors show a clear repertoire for the homocitrullinated Vimentin peptide (Vim116-135^Hcit^), with modification-specific and oligoclonal responses. Importantly, *in vivo* homocitrulline specific Vim116-135^Hcit,^Cyk8 371-388^Hcit^ and Aldo 140-157^Hcit^ responses are able to confer an anti-tumor effect in the murine B16 melanoma model. The Vim116-135^Hcit^ anti-tumor response was dependent upon tumor expression of MHC-II suggesting the direct recognition of PTMs on tumor is an important anti-tumor mechanism. Cancer patients also have a CD4 repertoire for Vim116-135^Hcit^. Together these results suggest that homocitrulline-specific immune responses can be generated in healthy mice and detected in human donors through a variety of HLA-restrictions. Immunization can induce responses to Vim116-135^Hcit,^Aldolase 140-157^Hcit^ and Cyk8 371-388^Hcit^ which provide anti-tumor therapy across several HLA-types. Our results advance our understanding of homocitrulline-specific immune responses, with implications for a number of fields beyond autoimmunity, including tumor immune surveillance.

## Introduction

Post translation modifications (PTM) are important for many cellular processes including altering the expression, function, signaling and cellular localization of proteins. PTMs also play a key role in epitope processing and presentation *via* MHC through altering protein cleavage (1, 2) increasing peptide processing and promoting epitope binding to MHC class I (MHC-I) or class II (MHC-II). Interestingly, they can also be induced as part of the antigen processing pathways (3–5). Inflammation or stress-related processes such as viral or oncogenic transformation of cells induces PTMs and an altered repertoire of MHC-associated peptides and has implications for many autoimmune-mediated diseases (6–9). Since the cellular stress associated with cancer cells can also lead to changes in PTMs (10). Therefore, inducing immune responses to PTMs will also have therapeutic applications in this field. PTMs which result in the generation of new epitopes that can be distinguished from native sequences include phosphorylation (11–13), glycosylation (14), deamidation of glutamine (8), citrullination (cit) of arginine (15–18) and homocitrullination (Hcit) of lysine (19). The latter three are particularly evident in autoimmunity where PTM epitopes are presented on MHC-II and recognized by modification specific CD4 T cells.

Homocitrullination, also known as carbamylation, results in the conversion of a positively charged amino acid lysine into a neutral amino acid homocitrulline. Like citrullination, this process can result in altered MHC binding properties (5). The conversion of lysine to homocitrulline is driven by the accumulation of cyanate (19, 20). In inflammatory conditions cyanate accumulation is driven by the myeloperoxidase (MPO) enzyme which is released by neutrophils (21). In addition to neutrophils, we have previously demonstrated a role for MPO-producing tumor-associated myeloid derived suppressor cells (MDSCs) in MPO production and ultimately in causing homocitrullination within the tumor microenvironment (22).

HLA restriction of T cell responses is important in determining the sensitivity of response to PTMs. Some HLA alleles have previously been identified as favoring the binding of PTM peptides and permitting stimulation of PTM-specific T cell responses. In the case of citrullination and deamidation of glutamine the HLA-DRB1 shared epitope and HLA-DQ2 or HLA-DQ8 have been shown to be favorable alleles, respectively (8, 23–25). We have recently shown that the HLA-DP4 allele also efficiently presents citrullinated epitopes (26). To date, there is little known regarding the HLA preference of homocitrulline-specific CD4 cells; although, due to overlap between citrulline and homocitrulline responses seen in patients, it is likely that homocitrullinated molecules are also presented by members of the HLA-DRB1 shared epitope alleles. Mouse MHC II from Balb/c and C57Bl/6 strains can mediate homocitrulline specific responses (19, 27), while for human HLA alleles, responses to the synthetic JED homocitrullinated peptide can be induced in transgenic HLA-DR4 mice (27). Furthermore, we have previously shown responses to several homocitrullinated peptides in transgenic HLA-HHDII/DP4 mice (22).

Here, we identify homocitrullinated peptides from three different antigens that stimulate homocitrulline-specific immune responses, restricted through multiple HLA-alleles. We characterized the response to Vim116-135^Hcit^ peptide and show stimulation of CD4 responses in Balb/c, HLA-HHDII/DR1 and HLA-DR4 transgenic mice and healthy human donors. We also show that these epitopes can induce effective tumor therapy in mouse models. Crucially, cancer patients have a repertoire that recognizes the Vim116-135^Hcit^ peptide, suggesting that immune responses to homocitrullinated peptides are not restricted to autoimmune disease. We suggest that the cross-restriction of homocitrullinated peptide specific CD4 responses may pave the way to designing an effective universal anti-tumor vaccine.

## Material and Methods

Unless otherwise stated reagents were obtained from Sigma-Aldridge and antibodies from eBioscience.

### Peptides

Peptides were analyzed for potential MHC-II binding using immune epitope database (IEDB) (http://www.iedb.org/) (28–30). Peptides (**Table 1**) were synthesized at >90% purity (Genscript), stored lyophilized at −80°C and then reconstituted in PBS on day of use.

**Table 1:** Peptide sequences and IEDB predicted binding scores.

Epitope	Homocitrullinated Sequence (with K replaced by Hcit)	IEDB predicted binding scores for wild type proteins Human MHC-II			IEDB predicted binding scores for wild type proteins Mouse-MHC II		
		DP4	DR1	DR4	I-Ed	I-Ab	I-Ad
**Aldolase**	**UniProt #P04075**						
Aldo74-93^Hcit^	IGGVILFHETLYQ-**hcit**-ADDGRP	2.51	33.91	30.22	18.75	56.5	50.0
Aldo140-157^Hcit^	**hcit**-DGADFA-**hcit-**WRCVL**-hcit**-IGEH	22.1	59.86	49.8	0.95	58.5	28.5
**Cyk8**	**UniProt #P05787**						
Cyk8 101-120^Hcit^	KFASFID-**Hcit**-VRFLEQQN-**Hcit**-MLE	0.97	12.27	5.04	3.9	67.5	74
Cyk8 112-131 ^Hcit^	LEQQN-**hcit**-MLET-**hcit**-WSLLQQQ-**hcit**-T	3.16	31.04	12.19	28.45	–	39.5
Cyk8 182-202 ^Hcit^	EIN-**hcit**-RTEMENEFVLI-**hcit-hcit**-DVDE	14.3	69.5	27.44	17	91.5	61
Cyk8 371-388^Hcit^	LREYQELMNV-**hcit**-LALDIEI	24.36	6.74	4.42	15.25	58.5	51.5
Cyk8 381-399 ^Hcit^	**hcit**-LALDIEIATYR-**hcit**-LLEGEE	18.10	39.89	12.39	14.5	82	38
**Vimentin**	**UniProt #P08670-1**						
Vim 116-135^Hcit^	NYID-**hcit**-VRFLEQQN-**hcit**-ILLAEL	2.12	11.76	5.06	20.2	56.0	31.55
Vim 215-235^Hcit^	LARLDLER-**hcit**-VESLQEEIAFL-**hcit**	21.67	76	24.51	21	76	43.5
Vim 255-275^Hcit^	QIDVDVS-**hcit**-PDLTAALRDVRQQ	82.48	58.03	17.21	31	18.5	35.5
Vim 286-303^Hcit^	EAEEWY-**hcit**-S-**hcit**-FADLSEAAN	2.06	18.99	4.76	9	21.4	36.5
Vim 431-460^Hcit^	LPLVDTHS-**hcit**-RTLLI-**hcit**-TVETRDGQV	35.09	24.07	9.5	16.45	74.5	24.5
**Hepatitis B**							
HepB 181-192	GFFLLTRILTIPQ						

Analysis is based on human sequences. All sequences used were homologous between mouse and humans with the exception of Cyk8 381-399^Hcit^ which has a T at position 389 in the murine sequence. Hcit, homocitrulline.

### Animals and Cell Lines

Animal experiments were carried out with ethical approval under a Home Office approved project license. HLA-DR4 mice (Model #4149, Taconic), HLA-A2/DR1 (HLA-HHDII/DR1, Pasteur Institute), HLA-A2.1+/+ HLADP4+/+ hCD4+/+ (HLA-HHDII/DP4; EM:02221, European Mouse Mutant Archive) transgenic mice and C57Bl/6J mice or Balb/c mice (Charles River) aged 8-12 weeks were used.

The murine melanoma B16F1 cell line (ATCC-CRL-6323) obtained from ATCC was engineered with mouse MHC K/O and transfected with HHDII (A2) alone or in combination with either constitutive HLA-DP4, HLA-DR1, HLA-DR4 or IFNγ-inducible HLA-DR4 (iDR4) using plasmids as previously described (17, 26). Cells were cultured in RPMI medium 1640 with L-glutamine (2 mmol/L), 10% FCS and appropriate antibiotics to maintain plasmids. Cell lines utilized were mycoplasma free, authenticated by suppliers (STR profiling), and used within ten passages.

### Immunization Protocol

Mice were injected subcutaneously (s.c.) with 25 μg of each peptide either as a pool or individually in combination with 5μg of CpG ODN 1826 (CpG) and Monophosphoryl Lipid A (MPLA) (InvivoGen) on days 1, 8 and 15 and responses analyzed on day 21.

For anti-tumor experiments, mice were implanted s.c. with tumor cells three days prior to immunization regime above. Implant doses were 5.0 x 10^5^ cells/mouse for B16 HHDII/DR1, 2.5x10^4^ cells/mouse for B16F1DR4 and 5x10^4^ cells/mouse for B16F1 iDR4 and B16F1 h2Ab1 B2M dKO cell lines (17, 31, 32). Tumor growth was monitored twice weekly and mice were humanely euthanized once tumors approach the license limit of 15 mm in diameter.

### ELISpot Assays

Murine ELISpot kits (Mabtech) were used with 5 × 10^5^ splenocytes/well and 10μg/ml synthetic peptide added to triplicate wells. 20μg/ml anti-CD8, or anti-CD4 antibodies were added to appropriate wells for blocking studies. Plates were incubated at 37°C for 40 hours in an atmosphere of 5% CO2. Example plate images are shown in **Supplementary Figure 1**. Spots were analyzed using an automated plate reader (Cellular Technologies Ltd).

### Peripheral Blood Mononuclear Cells (PBMC) Isolation and Processing

PBMCs were isolated from healthy donors and cancer patients (**Table 2**) using histopaque-1077 then depleted using anti-CD25 microbeads and MACS Cell Separation Columns (Miltenyi). Isolated CD25-depleted PBMCs were loaded with 5µM carboxyfluorescein succinimidyl ester (CFSE) (Thermofisher) (32). Cells were resuspended at 1.5×10^6^/ml in RPMI with autologous serum as previously described in full (32), plated and stimulated with vehicle, PHA (10 µg/ml), soluble anti-CD3 (positive control, 30ng/ml) or peptide (10µg/ml). Peptide concentration was selected based on reports in the literature using similar post-translationally modified epitopes (16, 18, 33). On day 10, 500µl of cells were stained with anti-CD4 (Thermofisher clone RPA-T4, 1/50 dilution) and anti-CD8 (Miltenyi Biotec clone REA734, 1/50 dilution) antibodies for 30 minutes and then samples were washed, fixed and analyzed immediately. For restimulation assays, cells were cultured in media with IL-2 IL-7 and IL-15 cytokines (Peprotech) and 10ug/ml of homocitrullinated peptides. On day 14, cells were harvested. Human IFNγ ELISpot kits (Mabtech) were used with 1 x 10^5^ cells/well with 10ug/ml peptide or PHA (10 µg/ml) in quadruplicate wells. Where appropriate, 7.6 µg/ml blocking antibodies (Isotype control clone MOPC-173 IgG2a kappa Biolegend, Anti-DR,DP,DQ clone Tu39 Biolegend, Anti-DP clone B7/21 Leinco, Anti-A,B,C cloneW6/32 Biolegend, Anti-DR clone G46-6 BD) were added for 30 minutes prior to peptides. ELISpot plates were incubated at 37°C for 24 hours in an atmosphere of 5% CO2 and then developed following the manufacturer’s instructions. Spots were analyzed using an automated plate reader (Cellular Technologies Ltd). All samples showed responses to PHA (data not shown).

**Table 2:** Healthy Donor information.

ID	Smoking status	HLA type							
**BD001**	Non-smoker	HLA A: *02, *32	HLA B *08,*44	HLA C: *04, *07	HLA DRB1: *03, *07	HLA DRB3: *01	HLA DRB4: *01	HLA DQA1: *02, *05 HLA DQB1: *01	HLA DPB1: *04
**BD002**	Non-smoker	HLA A: *02, *29	HLA B *44,*51		HLA DRB1: *04, *13	HLA DRB3: *01	HLA DRB4: *01	HLA DQB1: *03, *06	HLA DPB1: *04
**BD007**	Non-smoker	HLA A: *01, *32	HLA B *08, *15	HLA C: *07	HLA DRB1: *03, *13	HLA DRB3: *01, *03	–	HLA DQB1: *02, *06	HLA DPB1: *04, *13
**BD010**	Non-smoker	HLA A: *02, *11	HLA B *40, *44	HLA C: *03, *16	HLA DRB1: *13, *16	HLA DRB3: *03	HLA DRB5: *02	HLA DQB1: *05, *06	HLA DPB1: *04, *10
**BD014**	N/A	N/A							
**BD015**	Non-smoker	HLA A: *03, *24	HLA B: *07, *15	HLA C: *03, *07	HLA DRB1: *04, *15	HLA DRB4: *01	HLA DRB5: *01	HLA DQB1: *03, *06	HLA DPB1: *04
**BD016**	Ex-smoker	HLA A: *01, *02	HLA B: *8, *44	HLA C: *05, *07	HLA DRB1: *03, *15	HLA DRB5: *01	HLA DRB3: *02	HLA DQB1: *02, *06	HLA DPB1: *01, *04
**BD017**	Non-smoker	N/A							
**BD022**	Non-smoker	HLA A: *01, *02	HLA B: *35, *50	HLA C: *06, *12	HLA DRB1: *04, *07	–	–	HLA DQB1: *02, *03	HLA DPB1: *02, *04
**BD025**	Ex-smoker	HLA A *02, *29	HLA B *07, *57	HLA C: *06, *07	HLA DRB1: *01, *07	HLA DRB4: *01	–	HLA DQB1: *03, *05	HLA DPB1: *03, *13
**BD038**	Non-smoker	HLA A: *26, *33	HLA B: *40, *58	HLA C: *03	HLA DRB1: *09, *11	HLA DRB3: *02	HLA DRB4: *01	HLA DQB1: *03	HLA DPB1: *04, *05
**BD041**	Ex-smoker	HLA A: *01, *24	HLA B: *07, *40	HLA C: *03, *07	HLA DRB1: *04, *11	–	–	HLA DQB1: *03	HLA DPB1: *02, *04
**BD044**	Non-smoker	N/A							
**BD050**	Non-smoker	HLA A: *24, *26	HLA B *35, *34	HLA C: *04, *06	HLA DRB1: *07, *11	HLA DRB3: *02	HLA DRB4: *01	HLA DQA1: *02, *05 HLA DQB1: *02, *03	HLA DPB1: *02, *04
**BD051**	Non-smoker	HLA A: *11, *68	HLA B: *07, *15	HLA C: *05, *07	HLA DRB1: *11, *15	–	–	HLA DQB1: *06	HLA DPB1: *04, *19
**BD095**	Non-smoker	N/A							
**BD150**	Smoker	N/A							
**BD174**	Non-smoker	N/A							

N/A, data not available.

### TCR α and β Chains Analysis of Proliferating PBMCs

Stained CD4+/CFSE^high^ and CD4+/CFSE^low^ cell populations were bulk sorted into RNA protect (Qiagen) using a MoFlo XDP Sorter (Beckman Coulter). RNA was purified from sorted cells, RT-PCR performed, the cDNA was subjected to Amplicon rescued multiplex PCR (ARM-PCR) using human TCR α and β 250 PER primers (iRepertoire Inc., Huntsville, AL, USA) (26). Ten assessed sample libraries were pooled and sequenced using the Illumin//a MiSeq platform (Illumina, USA). Tree maps and D50 diversity values generated using iRweb software. Tree maps show each unique CDR3 as a colored rectangle, the size of each rectangle corresponds to each CDR3s abundance within the repertoire and the positioning is determined by the V region usage. Diversity was measured using D50 immune repertoire diversity index. The D50 index is a quantitative measure of the degree of diversity of T cells within a sample. The D50 is the percentage of T-cell clones that account for the cumulative 50% of the total CDR3s counted in the sample. The more diverse a library, the closer the value will be to 50. Low diversity values are associated with decreased diversity. Data are presented as non-normalized (which takes into account the frequency of each unique CDR3).

### Statistical Analysis

Statistical analysis was performed using GraphPad Prism software version 9. Comparative analysis of the ELISpot results was performed by applying paired or unpaired ANOVA or Student t test as appropriate with P values calculated accordingly. Comparison of tumor survival was assessed by log-rank test. Human responses were analyzed by Wilcoxon matched-paired test. P<0.05 values were considered statistically significant.

## Results

### Homocitrulline-Peptide Responses Can Be Induced Through Multiple HLA Alleles

**Figure 1:**
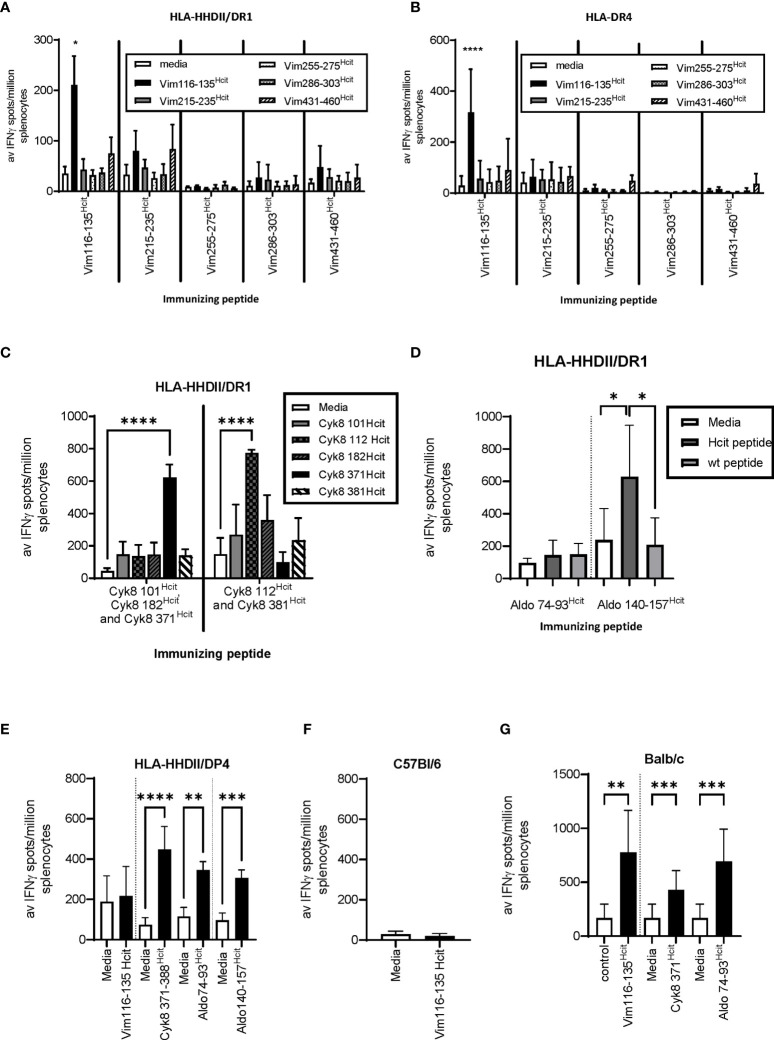
Homocitrullinated peptide responses in HLA-HHDII/DR1, HLA-DR4 and Balb/c mice. Mice were immunized with three doses of homocitrullinated peptides with the adjuvants CpG and MPLA. Responses to homocitrullinated vimentin (Vim) peptides were determined in HLA-HHDII/DR1 **(A)** and HLA-DR4 **(B)** mice. Responses to cytokeratin 8(Cyk8) peptides were determined in HLA-HHDII/DR1 mice **(C)**. Aldolase (Aldo) responses were screened in HLA-HHDII/DR1 mice **(D)**. Responding peptides were then also tested in HLA-HHDII/DP4 **(E)**, C57Bl/6 **(F)** and Balb/c **(G)** mice. For each study n≥3. Mean and standard deviations and significant p values are shown; ****p<0.0001, ***p<0.001, **p<0.01, *p<0.05.

We have previously shown that homocitrullinated peptides can stimulate responses restricted through the HLA-DP4 allele (22). In this study we first aimed to determine whether peptide immunization can induce homocitrulline-specific responses *via* HLA types other than the HLA-DP4 allele. The vimentin (Vim), cytokeratin 8 (Cyk8) and aldolase A (Aldo) sequences were analyzed by IEDB for sequences containing lysine within the predicted core binding region and since homocitrulline modification may influence the binding affinity, peptides with variable predicted binding scores for HLA-DR4 or HLA-DR1 alleles were selected. Predicted binding scores are shown in **Table 1**. Responses to five homocitrullinated peptides were assessed in HLA-transgenic mice expressing human HLA-DR1 along with chimeric human HLA-A2 (HHDII) or HLA-DR4 (**Figures 1A, B**). This screening identified significant responses to Vim116-135^Hcit^ in both strains. The homocitrullinated peptide Vim116-135^Hcit^ was synthesized with the lysine residues at positions 120 and 129 both converted to homocitrulline (**Table 1**).

Responses were also screened for five Cyk8 peptides (**Figure 1C**) and two Aldo peptides (**Figure 1D**) in HLA-transgenic mice expressing human HLA-DR1 and chimeric HLA-A2 (HLA-HHDII/DR1) mice. This screening identified Cyk8 371-388^Hcit,^ Cyk8 112-131^Hcit^ and Aldo140-157^Hcit^ as inducing immune responses in the HLA-HHDII/DR1 mice. Cyk8 371-388^Hcit^ was synthesized with the lysine converted to homocitrulline at position 381 and Aldo140-157^Hcit^ contained three homocitrulline residues at positions 140, 147 and 153 (**Table 1**).

We have previously observed that both Aldo140-157^Hcit^ and Cyk8 371-388^Hcit^ can induce immune responses in HLA-HHDII/DP4 mice (22) suggesting an additional HLA restriction. The peptides were therefore tested in additional mouse strains. In HLA-HHDII/DP4 mice, the previously described responses were seen for Cyk8 371-388^Hcit^, Aldo74-93^Hcit^ and Aldo140-157^Hcit^ (**Figure 1E**). No immune responses to Vim116-135^Hcit^ were observed in HLA-HHDII/DP4 (**Figure 1E**) nor C57Bl/6 mice (**Figure 1F**). However, significant Vim116-135^Hcit^ (p=0.0018), Cyk8 371-388^Hcit^ (p=0.0005) as well as Aldo 74-93^Hcit^ (p=0.0001) responses were seen in Balb/c mice (**Figure 1G**).

This data shows that immunization with homocitrullinated peptides can induce responses in mice expressing the shared epitope human HLA alleles DR4 and DR1 as well as human HLA-DP4 and murine H-2d haplotype. However, cross reactivity between HLA alleles varies between peptides.

### Homocitrulline Responses Are Modification Specific and CD4 Mediated

**Figure 2:**
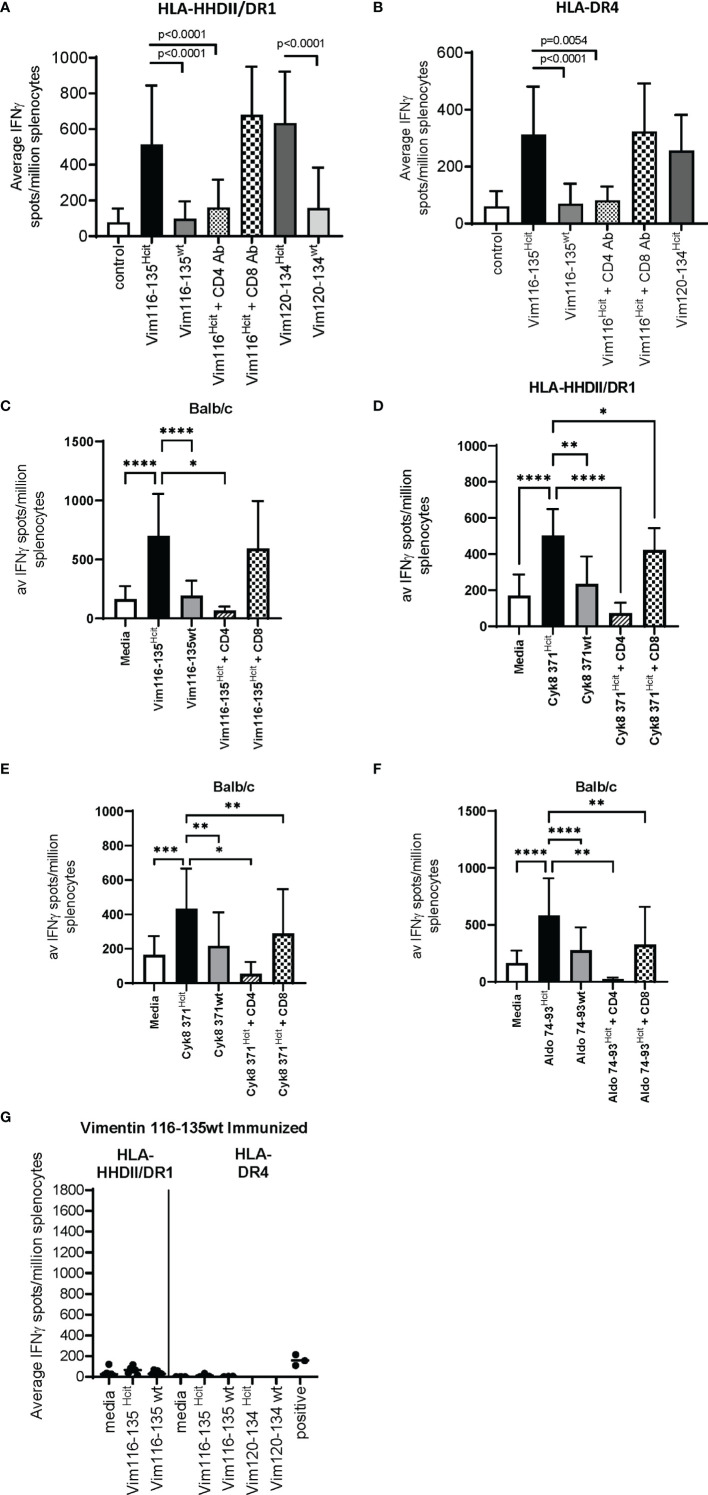
Characterization of the homocitrulline-mediated immune responses. Mice were immunized with homocitrullinated peptides then assessed for responses using IFNγ ELISpot assays. ELISpots were then performed with homocitrullinated (Hcit) and wild type (wt) peptides or homocitrullinated peptides in the presence of anti-CD4 or anti-CD8 blocking antibodies. Vim116-135^Hcit^ responses were assessed in HLA-HHDII/DR1 **(A)**, HLA-DR4 **(B)** and Balb/c **(C)** mice. Cyk8 371-388^Hcit^ responses were assessed in HLA-HHDII/DR1 **(D)** and Balb/c **(E)** mice. Aldo 74-93^Hcit^ responses were only assessed in Balb/c mice **(F)**. Mice were also immunized with wild type Vim116-135 peptide and responses were assessed in HLA-HHDII/DR1 and HLA-DR4 mice **(G)**. For each study n≥3, Homocitrulline immunized mice were assessed in ≥3 independent studies. Mean and standard deviations and significant p values are shown; ****p<0.0001, ***p<0.001, **p<0.01, *p<0.05.

Immunization with long peptides has the potential to stimulate both CD4 and CD8 responses, therefore we further characterized the immune responses to three peptides to determine if responses were CD4 mediated and homocitrulline specific. Mice were immunized with the Vim116-135^Hcit^ peptide and immune responses determined by IFNγ ELISpots. In HLA-HHDII/DR1 (**Figure 2A**), HLA-DR4 (**Figure 2B**) and Balb/c (**Figure 2C**) mice responses to the modified homocitrulline peptide were significantly greater than the responses to the wild type (wt) peptide (p<0.0001 for all strains). Anti-CD4 blocking antibodies significantly reduced the IFNγ response to Vim116-135^Hcit^ (p<0.0001, p=0.0054 and p=0.0143 for HLA-HHDII/DR1, HLA-DR4 and Balb/c respectively) but anti-CD8 antibody had no significant effect on the immune response. Example ELISpot images are shown in **Supplementary Figure 1**.

The Cyk8 371-388^Hcit^ response in HLA-HHDII/DR1 (**Figure 2D**) and Balb/c (**Figure 2E**) mice was also assessed. IFNγ responses to Cyk8 371-388^Hcit^ showed minimal cross reactivity to the wt peptide with the Hcit responses significantly greater than the wt response in both strains (p=0.0045 and p=0.0073 for HLA-HHDII/DR1 and Balb/c respectively). In both strains the IFNγ response to Cyk8 371-388^Hcit^ was partially blocked by anti-CD8 antibodies but fully blocked by anti-CD4 antibodies. In HLA-HHDII/DR1 mice responses were reduced from a mean of 503 spots/million splenocytes to 73 spots/million splenocytes (p<0.0001) after blocking with the anti-CD4 antibody and only slightly reduced to a mean of 423 spots/million splenocytes in responses to anti-CD8 blocking antibodies (p=0.0148). In Balb/c mice the mean response of 433 spots/million splenocytes was reduced to 53 spots/million splenocytes after CD4 blocking (p=0.0117) and 289 spot/million splenocytes after CD8 blocking (p=0.0048), suggesting that the Cyk8 371-388^Hcit^ responses were also CD4 and CD8 mediated.

Finally, the Aldo74-93^Hcit^ response was characterized in Balb/c mice (**Figure 2F**). IFNγ responses to Aldo74-93^Hcit^ were significantly greater than wild type responses (p<0.0001). The Aldo74-93^Hcit^ response was partially blocked by anti-CD8 antibodies but fully blocked by anti-CD4 antibodies. Responses were reduced from a mean of 583 spots/million splenocytes to 24 spots/million splenocytes (p=0.0020) after blocking with the anti-CD4 antibody and partially reduced to a mean of 255 spots/million splenocytes in responses to anti-CD8 blocking antibodies (p=0.0059).

To further confirm that the Vim116-135^Hcit^ response is modification specific immunizations with the wild type Vim116-135wt peptide were performed (**Figure 2G**). No immune responses to Vim116-135^Hcit^ were observed in either HLA-HHDII/DR1 or HLA-DR4 mice. This confirms that only the modified peptide can induce an immune response.

Together these results confirm that the immune responses to Vim 116-135^Hcit^, Cyk8 371-388^Hcit^ and Aldo 74-93^Hcit^ were CD4 and possibly CD8 mediated and homocitrulline specific in mice with the appropriate MHC alleles.

### Humans Have a Repertoire That Can Recognize the Vim116-135^Hcit^ Peptide

Having demonstrated that immunized transgenic mice expressing human HLA types show homocitrulline-specific immune responses, we next determined whether healthy human donors exhibit a repertoire for homocitrullinated peptides. For this we concentrated on the Vim116-135^Hcit^ peptide.

**Figure 3:**
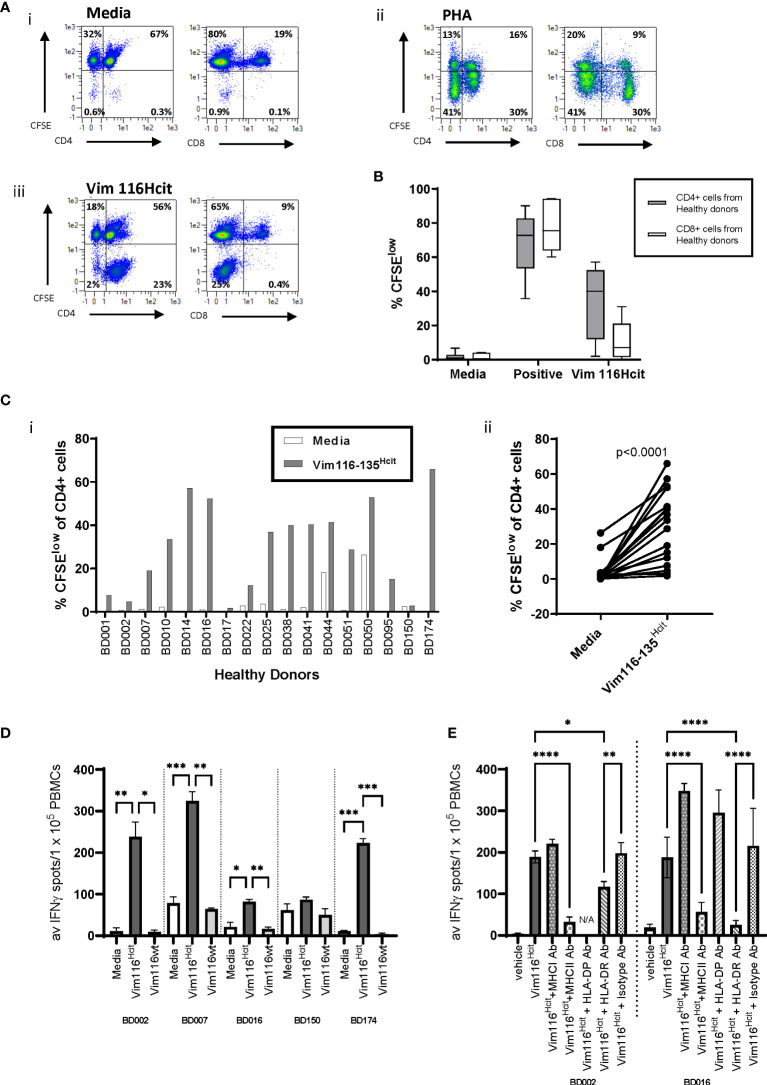
Heathy Human Donors have a repertoire that can recognize the Vim116-135^Hcit^ peptide. PBMCs were isolated from 17 healthy donors and assessed for proliferative responses to homocitrullinated peptides using a CFSE dilution assay. Example plots from healthy donor BD0051 **(A)** show proliferation (CFSE) vs CD4 or CD8 in media control **(i)** PHA stimulated **(ii)** or Vim116-135^Hcit^ stimulated **(iii)** cells after gating on the total lymphocyte population. CFSE proliferation in the gated CD4 or CD8 populations was assessed **(B)**. Given that the majority of proliferation in response to Vim116-135^Hcit^ was found in the CD4+ population, the percentage of the CD4+ proliferation was assessed in individual healthy donors **(Ci)** and paired analysis comparing media control to peptide stimulation **(Cii)**. Restimulation assays were performed on PMBCs from 5 healthy donor that had been cultured with Vim116-135^Hcit^ peptide for 14 days. IFNγ ELISpot showed restimulation responses to homocitrullinated (hcit) and wild type (wt) peptides **(D)**. Restimulation to Vim116-135^Hcit^ was also performed for two donors in the presence of anti-MHC-I, MHC-II, DP, DR or isotype blocking antibodies **(E)**. Summary data with significant p values are shown; ****p<0.0001, ***p<0.001, **p<0.01, *p<0.05.

PBMCs were isolated from 17 healthy donors and assessed for proliferative responses to homocitrullinated peptides. Donor details including HLA-types are shown in **Table 2**. Flow cytometry staining was used to assess CFSE proliferation in the CD4+ or CD8+ cell populations. Proliferation was assessed at day 10 post-stimulation as this timepoint showed clear peptide-induced proliferative responses. An example staining profile is shown in **Figure 3A** and additional example CFSE profiles for day 7 and day 10 are shown in **Supplementary Figure 2**. CFSE proliferation to the positive control PHA was seen in both the CD4 and CD8 populations, however, Vim116-135^Hcit^ proliferation was predominantly seen in the CD4 population. Collated data from healthy donors is shown in **Figure 3B** whilst CD4 proliferative responses in individual healthy donors are shown in **Figure 3Ci**. In PBMCs from healthy donors, 15/17 showed CD4 T-cell proliferation in response to Vim116-135^Hcit^ peptide. Across all donors, Vim116^Hcit^ induced a significant increase in CD4 proliferation compared to media only (p<0.0001) (**Figure 3Cii**).

To determine whether the Hcit responses seen in healthy donors were modification-specific restimulation assays were performed on 5 healthy donor PMBCs that had been cultured with modified peptide for 14 days (**Figure 3D**). IFNγ ELISpot showed restimulation responses to modified but not wt peptide. All responding donors showed a significantly higher response to Vim116-135^Hcit^ compared to Vim116-135wt peptide. For two donors restimulation assays were also performed in the presence of blocking antibodies (**Figure 3E**). In both donors’ responses to Vim116-135^Hcit^ were significantly reduced by addition of pan-MHC-II blocking antibodies or anti-HLA-DR blocking antibodies indicating the involvement of the HLA-DR allele. Together this data confirms that healthy human PBMCs have a modification specific repertoire for this peptide that is likely restricted through HLA-DR alleles.

### The Human Responses to Vim116-135^Hcit^ Are Oligoclonal

**Figure 4:**
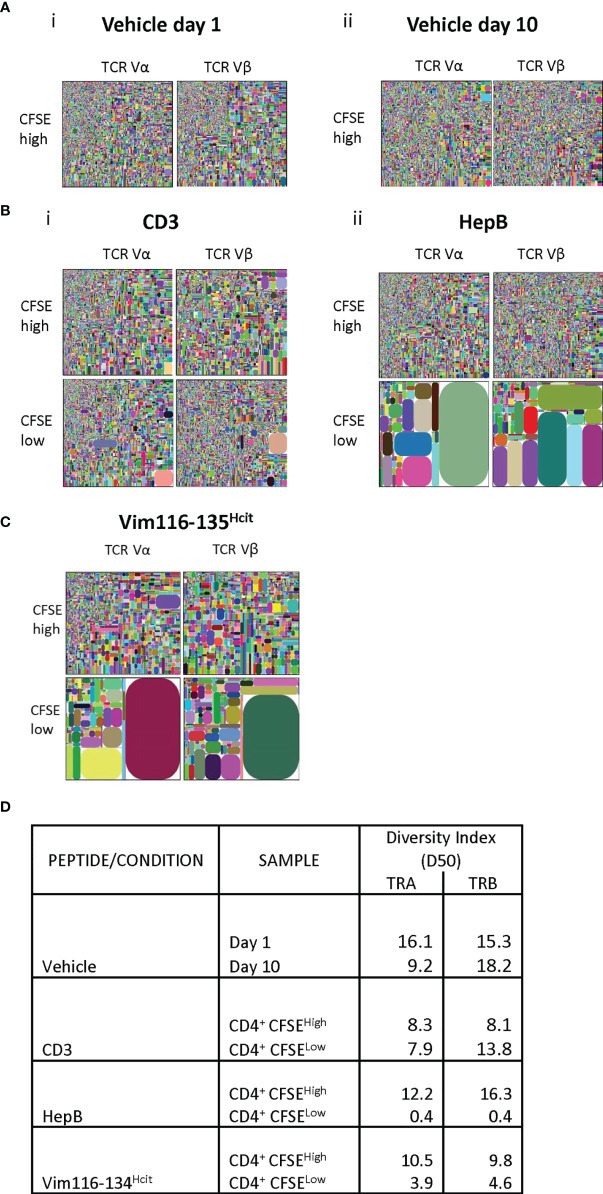
Human response to Vim116-135^Hcit^ is oligoclonal. TCR α and β repertoire diversity was assessed in the CD4+ CFSE^high/low^ cells in one healthy donor (BD016). Responses to vehicle **(A)**, positive control CD3 and HepB **(B)** or Vim116-135^Hcit^ peptide stimulation **(C)** were assessed. Tree maps depict TCR α and β chain CDR3 clonotype usage in relation to repertoire size. Each rectangle represents a unique CDR3 nucleotide sequence and rectangle size denotes the relative frequency of an individual sequence. Colors are randomly assigned and thus do not represent the same CDR3 sequence between plots. Diversity Index for each population is shown in **(D)**.

To determine the clonality, TCR Vα and Vβ sequencing was performed on proliferating and non-proliferating CD4 T-cells isolated from healthy donor that had been stimulated *ex vivo.* TCRα and β CDR3 tree maps were used to display clonality (**Figure 4**). Stimulation with vehicle showed minimal proliferative responses and no consistent differences in the clonality of the non-proliferative population (**Figure 4A**). Polyclonal CD3 stimulation also led to no clear changes in the diversity of clonotypes, whereas responses to the HLA-DP4 restricted Hepatitis B peptide showed decreased diversity after proliferation (**Figure 4B**). Vim116-135^Hcit^ stimulated samples also showed decreased CDR3 diversity in the proliferating CD4+CFSE^low^ cells with a dramatic increase in the relative frequency of subsets of CDR3 sequences suggesting a focused repertoire of cells responding to Vim116-135^Hcit^ (**Figure 4C**). The oligoclonal nature of the proliferating CD4 T cells was confirmed by the lower diversity index (D50) of the CDR3 sequences from the proliferating (CD4^+^CFSE^low^) compared to non-proliferating (CD4^+^CFSE^high^) populations (**Figure 4D**).

### Vim116-135^Hcit^ Immunization Is Associated With Improved Anti-Tumor Response *In Vivo*

**Figure 5:**
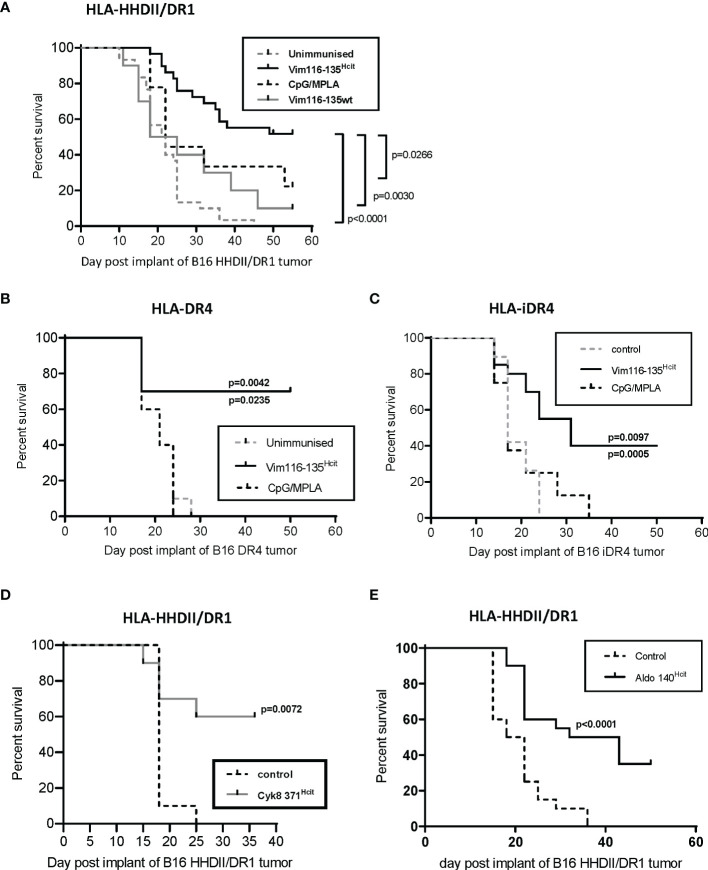
Vim116-135^Hcit^ responses are associated with an anti-tumor response. HLA-HHDII/DR1 **(A)** and HLA-DR4 **(B)** mice were implanted with HLA-matched B16 F1 cells expressing MHC-II under a constitutive **(A, B, D, E)** or inducible **(C)** promoter on day 1. On day 4, 11 and 18 mice were immunized with homocitrullinated (Hcit) or wild type (wt) Vim116-135 peptides or adjuvant only controls. Anti-tumor survival was compared to unimmunized control mice. Anti-tumor studies were also performed with Cyk8 371^Hcit^ **(D)** and Aldo140-157^Hcit^ **(E)** in HLA-HHDII/DR1 mice implanted with HLA-matched B16 cells. For each group and study n≥10, apart from CpG/MPLA control in panel B where n=5. significant p values are shown.

We have identified HLA DR-allele associated homocitrulline-specific immune responses. Our previous studies have shown that MDSC-produced MPO can mediate homocitrullination in the tumor microenvironment making homocitrullinated epitopes good targets for tumor therapy (22). However, not all homocitrulline-specific immune responses show anti-tumor responses suggesting that not all lysines are naturally homocitrullinated or presented. Therefore, we next determined if the immune response to Vim116-135^Hcit^ was sufficient to provide tumor therapy *in vivo*. Transgenic mice were implanted with HLA-matched B16 F1 cells and immunized with Vim116-135^Hcit^ peptide. Immunization schedule is presented in **Supplementary Figure 3A**. In mice containing the HLA-HHDII/DR1 allele (**Figure 5A**), Vim116-135^Hcit^ immunization provided significantly better survival than mice from the unimmunized control (p<0.0001), Vim116-135wt peptide immunized (p=0.0030) or CpG/MPLA adjuvant only (p-=0.0266) groups. Tumor growth curves are presented in **Supplementary Figure 3B**. In HLA-DR4 mice (**Figure 5B**) Vim116-135^Hcit^ immunization was also associated with a significant increase in survival when compared to unimmunized control mice (p=0.0042) or CPG/MPLA control (p=0.0235).

Since, both these cell lines were engineered to express MHC-II constitutively and expression of MHC-II in non-APCs, including most cancer cells, is not constitutive but driven by the IFNγ-induced CIITA promoter pIV (34) we engineered B16 cells to express transgenic MHC-II under an IFNγ-inducible promoter (iDR4. Mice implanted with B16 iDR4 (**Figure 5C**) and immunized with Vim116-135^Hcit^ showed a significant survival advantage over mice immunized with adjuvant only (p=0.0097) or unimmunized mice (p=0.0005). Tumor growth curves are shown in **Supplementary Figure 3C**. Together this data shows that immunization with Vim116-135^Hcit^ confers a potent anti-tumor response.

To confirm whether other Homocitrullinated peptides can also induce an anti-tumor response the Cyk8 371-388^Hcit^ and Aldo 140-157^Hcit^ peptides were also tested *in vivo*. In HLA-HHDII/DR1 mice implanted with B16 HHDII/DR1 tumor cells immunization with Cyk8 371-388^Hcit^ induced a significant survival advantage when compared to unimmunized control (p=0.0072) (**Figure 5D**). In this same model, immunization with Aldo 140-157^Hcit^ also resulted in a significant anti-tumor effect (p<0.0001) when compared to unimmunized control (**Figure 5E**).

### Expression of MHC II Is Critical for the Vim116-135^Hcit^ Induced Anti-Tumor Response

**Figure 6:**
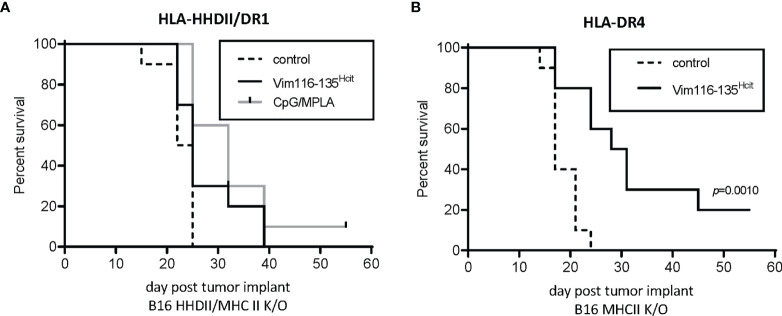
Anti-tumor response requires direct presentation on MHC-II by tumor. Anti-tumor studies were performed using tumor which expressed MHC-I but were knocked out for MHC-II. HLA-HHDII/DR1 **(A)** and HLA-DR4 **(B)** mice were challenged with B16 K/O MHC-II cell lines on day 1. On day 4, 11 and 18 mice were immunized with Vim116-135^Hcit^ peptide or adjuvant only controls. Anti-tumor survival was compared to unimmunized control mice. For each group n=10, significant p values are shown.

CD4 T cells recognize homocitrullinated epitopes but the anti-tumor effect may be mediated by a bystander effect of CD4 activation of other effector cells such as CD8, NK cells or macrophages or, CD4s may act directly *via* induction of MHC-II on tumor cells. We therefore investigated if the expression of MHC-II on tumor cells was required for the efficacy of the vaccine. HLA-HHDII/DR1 mice were implanted with B16 cells expressing matched MHC-I but no MHC-II (B16 HHDII/MHC-I/II KO). In this model APCs express MHC-II and CD8 T-cells can recognize the tumor *via* MHC-I. The anti-tumor effect of Vim116-135^Hcit^ peptide immunization was completely lost (**Figure 6A**) in this model, suggesting therapy was not mediated by other effector cells. In contrast, HLA-DR4 mice implanted with B16 MHC-II K/O cells and immunized with Vim116-135^Hcit^ (**Figure 6B**). did demonstrate a significant survival advantage over control unimmunized mice (p=0.0010). HOWEVER, this advantage was still reduced compared to the model expressing MHC-II shown **Figure 5B** an suggesting that, in this case, both bystander and direct CD4 recognition of tumor may play a role in the vaccine efficacy(p=0.0010).

### Cancer Patients Have a Repertoire That Can Recognize the Vim116-135^Hcit^ Peptide

**Figure 7:**
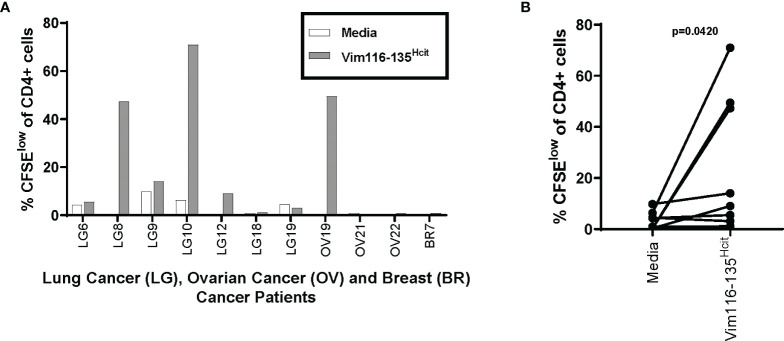
Cancer Patients also have a repertoire that can recognize the Vim116-135^Hcit^ peptide. PBMCs were isolated from 11 cancer patients (7 Lung cancer patients, 2 Ovarian patients and 1 Breast cancer patient) and assessed for proliferative responses by CFSE dilution assay to homocitrullinated peptide. Graph shows CD4 proliferation in individual patients **(A)** and paired analysis comparing media control to Vim16-135^Hcit^ stimulation **(B)**.

Given that Vim116-135^Hcit^ shows a strong anti-tumor response *in vivo* in mouse models, we next determined if cancer patients had an immune repertoire capable of responding to this peptide. PBMCs were isolated from seven lung cancer patients, two ovarian cancer patients and one breast cancer patient (n=11, **Table 3**) and assessed for proliferative responses to Vim116-135^Hcit^. PBMCs from 4/11 cancer patients showed responses to Vim116-135^Hcit^ (**Figure 7A**). Across all donors, Vim116-135^Hcit^ induced a significant increase in CD4 proliferation compared to media only (p=0.0420) (**Figure 7B**). This suggests that some cancer patients may be able to respond to immunization making this a potential anti-cancer therapy. Overall, this study has identified three homocitrullinated peptides that have different but overlapping HLA-restrictions. The responses are modification specific and CD4-mediated. The results suggest that these responses can be utilized to target tumors and that they can induce responses in healthy donors and cancer patients.

**Table 3:** Cancer patient information.

ID	Smoking status	Indication/Treatment
**LG6**	Ex-smoker	Lung adenocarcinoma/currently none, previous chemotherapy and checkpoint inhibitors
**LG8**	Ex-smoker	Lung adenocarcinoma/Tyrosine Kinase inhibitor and Steroid
**LG9**	Ex-smoker	Lung adenocarcinoma/Chemotherapy
**LG10**	Smoker	Lung adenocarcinoma/Checkpoint inhibitor
**LG12**	Ex-smoker	SCLC/Chemotherapy
**LG18**	Ex-smoker	Lung adenocarcinoma/treatment naïve
**LG19**	Ex-smoker	Lung adenocacinoma/checkpoint inhibitor
**OV19**	Unknown	Ovarian serous adenocarcinoma (low grade)/post surgery and pre systemic treatment
**OV21**	Unknown	Ovarian serous adenocarcinoma/Finished chemotherapy
**OV22**	Unknown	Peritoneal adenocarcinoma/treatment naïve
**BR7**	Unknown	Triple negative breast cancer

Lung (LG), Ovarian (OV) and Breast (BR) cancer patients.

## Discussion

The stress associated PTMs citrullination (cit) and homocitrullination are known to produce autoantigens in some disease situations. Autoreactive antibodies and T cells that recognize homocitrullinated epitopes have been described alongside citrullinated epitopes in rheumatoid arthritis (RA) (35–39). Anti-Hcit responses have also been seen in other autoimmune conditions such as systemic lupus erythematosus (SLE) (9, 40). Expansion of the repertoire of T cells responding to post-translationally modified peptides is believed to contribute to disease severity (41, 42). In RA responses have been linked to expression of the shared epitope (SE) MHC-II alleles (43). SE alleles are DRB1 molecules including DR4 and DR1 with a shared amino acid motif that predisposed to RA (44, 45). The SE can act as a signal transduction ligand to increase the abundance of citrullinated peptides and bone damage (46). Although interestingly, Lac *et al.* showed in transgenic mice that while anti-cit responses were confined to the shared epitope, anti-Hcit responses can be induced outside of the shared epitope alleles (27). These findings have resulted in Hcit modifications being known primarily as a side effect of inflammation that leads to aberrant immune responses and the breaking of tolerance.

In this study we have shown that normal human donors have a repertoire of CD4 T cells that can respond to Hcit peptides. This anti-Hcit response was observed in both SE and other HLA types. Human data demonstrated that healthy donors with a variety of HLA types showed immune responses to Vim116-135^Hcit^. In fact, characterization of the restimulation response in donor BD016 showed a donor that does not express DR4 or DR1 alleles can induce a DR-dependent, Hcit specific immune response. Data from our previous study shows that responses in healthy donors are also seen to other Hcit peptides (22). These healthy donors have no diagnosis of RA despite the presence of Hcit-reactive T cells in the majority of donors tested.

This study also showed that in human SE allele transgenic and non-SE allele Balb/c mice modification specific Hcit T cell responses can be induced *via* immunization. In mice, responses were seen in HLA-DR4 and HLA-DR1 transgenic strains but were also observed in Balb/c mice. Other studies have also shown responses in Balb/c and C57Bl/6 mice and transgenic mice expressing the non-SE allele HLA-DP4 (19, 22, 27). This confirms that the ability to differentiate between homocitrullinated and wild type peptides is not a phenomenon restricted to the SE allele but is in fact integrated into several MHCII alleles across species.

These results align with the hypothesis that presentation of homocitrullinated epitopes is a normal response to cellular stress, inflammation and tissue damage. The accumulation of homocitrullinated proteins has been associated with normal aging (47). Homocitrullination of specific proteins are also known to have physiological effects. For example, homocitrullination of haemoglobin plays a role in the response to hypoxia and homocitrullination of erythropoietin maintains neuroprotective effects without any erythropoiesis (48). Homocitrullination can result from the accumulation of urea (21) but in inflammatory conditions, hydrogen peroxide breakdown by the myeloperoxidase (MPO) enzyme leads to the conversion of thiocyanate to cyanate and isocyanic acid which is turn results in homocitrullination of lysine residues (21, 49). MPO driven homocitrullination has been associated with inflammation and smoking (49, 50). Studies in fibroblasts show that exposure to cyanate or urea can drive accumulation of intracellular homocitrullinated peptides and that these modified peptides can then be removed *via* proteasomal degradation (51). As seen in the context of autoimmune disease, homocitrullination of proteins can alter proteolytic cleavage and lead to MHC presentation of neoepitopes (27, 38, 52). However, the presence of diverse alleles that can recognize Hcit-specific peptides and the widespread presence of homocitrullinated peptides leads us to believe that this system may have a role in normal cell surveillance (10).

The theory of immune surveillance suggests that the immune system can recognize and kill some precancerous cells (53–55). However, in established tumors the tumor microenvironment (TME) is highly evolved to suppress pro-inflammatory responses and therefore the immune response is attenuated. The success of checkpoint inhibitors is to re-establish immune attack in order to eliminate cancer cells. In the TME chronic cellular stress occurs, and there can be high levels of myeloid derived suppressor cells (MDSCs). We have previously shown that the MDSCs can provide a source of MPO and drive homocitrullination (22). Tumor cells can also produce high levels of H_2_O_2_, required for MPO driven homocitrullination, as a result of the oncogenic transformation associated with antioxidant imbalances (56). Therefore, vaccination to recover and expand pro-inflammatory Hcit specific T cell responses may be highly effective in some tumors. In this study we showed that Hcit-peptide immunization in Balb/c and HLA transgenic mice can provide tumor therapy. The responses were dependent on the tumors presenting peptide *via* MHCII showing the importance of direct tumor recognition and inflammation in upregulating MHC-II. Our previously published work demonstrates that increased immune infiltrate is correlated with successful tumor regression following homocitrullinated peptide vaccination (22). However, future studies *in vivo* and in patients should seek to further characterized the tumor microenvironment following immunization with homocitrullinated peptides including the peptides discussed in this paper. Interestingly we also demonstrate that unimmunized cancer patients have a repertoire of cells that can recognize the Vim116-135^Hcit^ peptide. Despite the detection of responding CD4 T cells, there is some evidence that the cancer patients showed a lower response rate to this peptide (4/11) than healthy donors (15/17) suggesting these responses have been switched to a regulatory phenotype. Clinical trials will need to be carried out to determine whether immunization can reverse the suppression or stimulate a *de novo* response.

In contrast to the situation in cancer where an immunosuppressive tumor environment is prevalent, some conditions including those seen in RA patients, lead to chronic inflammation and autoimmune disease. These aberrant responses may be exacerbated in individuals with an SE allele due to better binding of modified peptides to HLA or other pro-inflammatory factors. One mouse study showed that immunization with Hcit peptides induced Hcit and cit responses in HLA-DR4 transgenic mice but only Hcit responses in C57Bl/6 mice providing further evidence that HLA alleles effect the type of immune responses generated (27). SE alleles DRB1*0401 and *0402 transgenic mice were shown to be predisposed to inducing Th17 responses which are effective at removing acute infection but that can drive autoimmune disease in the presence of chronic inflammation (57). However, Balb/c mice exhibited signs of RA disease after immunization with homocitrullinated peptides suggesting autoimmune disease is not completely restricted to individuals with SE alleles (19). The situation is complicated and the conditions which drive pro-inflammatory disease are not fully understood. Analysis of RA patients and matched first degree relatives without inflammation showed that both groups have similar levels of anti-Hcit antibodies, suggesting that homocitrulline targeted immunity alone is not always associated with disease (58). However, these autoimmune responses will need to be monitored extensively in any clinical trials aiming to use homocitrullinated peptide vaccination as an anti-tumor therapy. It may mean autoimmune disease would be a primary exclusion criterion for trials and that levels of autoantibodies would need to be continually measured in all patients.

Overall, this study identifies Vim116-135^Hcit^ as a peptide that can induce Hcit-specific CD4 T cell responses in healthy humans and mice. It also identified a number of other homocitrullinated peptides that can stimulate immune responses *via* various HLA types. *In vivo* studies show that these responses can provide a strong anti-tumor effect that is dependent on tumor expression of MHCII. The results contribute to our understanding of Hcit as a cellular mechanism beyond the scope of autoimmune disease and may open exciting new avenues for the treatment of cancer in a diverse HLA-background.

## Data Availability Statement

The raw data supporting the conclusions of this article will be made available by the authors, without undue reservation.

## Ethics Statement

The studies involving human participants were reviewed and approved by Research Ethics Committee, University of Nottingham. The patients/participants provided their written informed consent to participate in this study. The animal study was reviewed and approved by University of Nottingham ethics committee Nottingham Trent university ethics committee.

## Author Contributions

LD and VB directed the study. KC, WX, SA, PS, AA, SS, RC, and ID performed experiments and analyzed data. RM provided reagents and support. KC, WX, and VB designed experiments, analyzed the data and wrote the paper. All authors contributed to the article and approved the submitted version.

## Funding

This study received funding from Scancell Ltd. The funder was involved in the study design, collection, analysis, interpretation of data, the writing of this article and the decision to submit it for publication.

## Conflict of Interest

KC, WX, VB, and LD have ownership interest in a patent WO2020053304A2. LD is a director and shareholder in Scancell Ltd. All authors are employees of Scancell Ltd.

This study received funding from Scancell Ltd. The funder had the following involvement with the study: design, data collection and analysis, decision to publish and preparation of the manuscript.

## Publisher’s Note

All claims expressed in this article are solely those of the authors and do not necessarily represent those of their affiliated organizations, or those of the publisher, the editors and the reviewers. Any product that may be evaluated in this article, or claim that may be made by its manufacturer, is not guaranteed or endorsed by the publisher.

## Supplementary Material

Supplementary Figure 1 Example ELISpot images. Example ELISpot images show HLA-HHDII/DR1 mice were immunized with the Vim116-135^Hcit^ peptide. Responses for individual mice were assessed in triplicate to media only control, 10ug/ml peptides or positive control LPS. Responses to peptide were also assessed in the presence of anti-CD4 or anti-CD8 blocking antibodies.

Supplementary Figure 2 Additional CFSE example plots. PBMCs were isolated from healthy donors and assessed for proliferative responses to homocitrullinated peptides using a CFSE dilution assay. Example plots from healthy donor BD0051 at day 7 **(A)** show proliferation (CFSE) vs CD4 or CD8 in media control (i) PHA stimulated (ii) or Vim116-135^Hcit^ stimulated (iii) cells after gating on the total lymphocyte population. Example CFSE proliferation profiles for donor BD051 on day 7 and day 10 in the gated CD4 or CD8 populations **(B)**. Peptide responses were superior on day 10.

Supplementary Figure 3 Tumor growth curves. For all tumor studies mice were implanted with tumor on day 1 and then immunized on days 4, 11 and 18 **(A)**. For some mice the home office license agreed humane end point was reached prior to final immunization and mice were terminated. HLA-HHDII/DR1 **(B)** and HLA-DR4 **(C)** mice were implanted with HLA-matched B16 F1 HHDII/DR1 **(B)** or HLA-iDR4 **(C)** cells. Mice were immunized with homocitrullinated (Hcit) or wild type (wt) Vim116-135 peptides or adjuvant only controls. Tumor growth for each group is shown. Boxed number represents the number of mice that were tumor-free at the end of the study for each group.
