# Supplementary figures and images for: Vaccine Can Induce CD4-Mediated Responses to Homocitrullinated Peptides via Multiple HLA-Types and Confer Anti-Tumor Immunity
Source: Front Immunol. 2022 Apr 8;13:873947. doi: 10.3389/fimmu.2022.873947 (PMC9028767; doi:10.3389/fimmu.2022.873947)

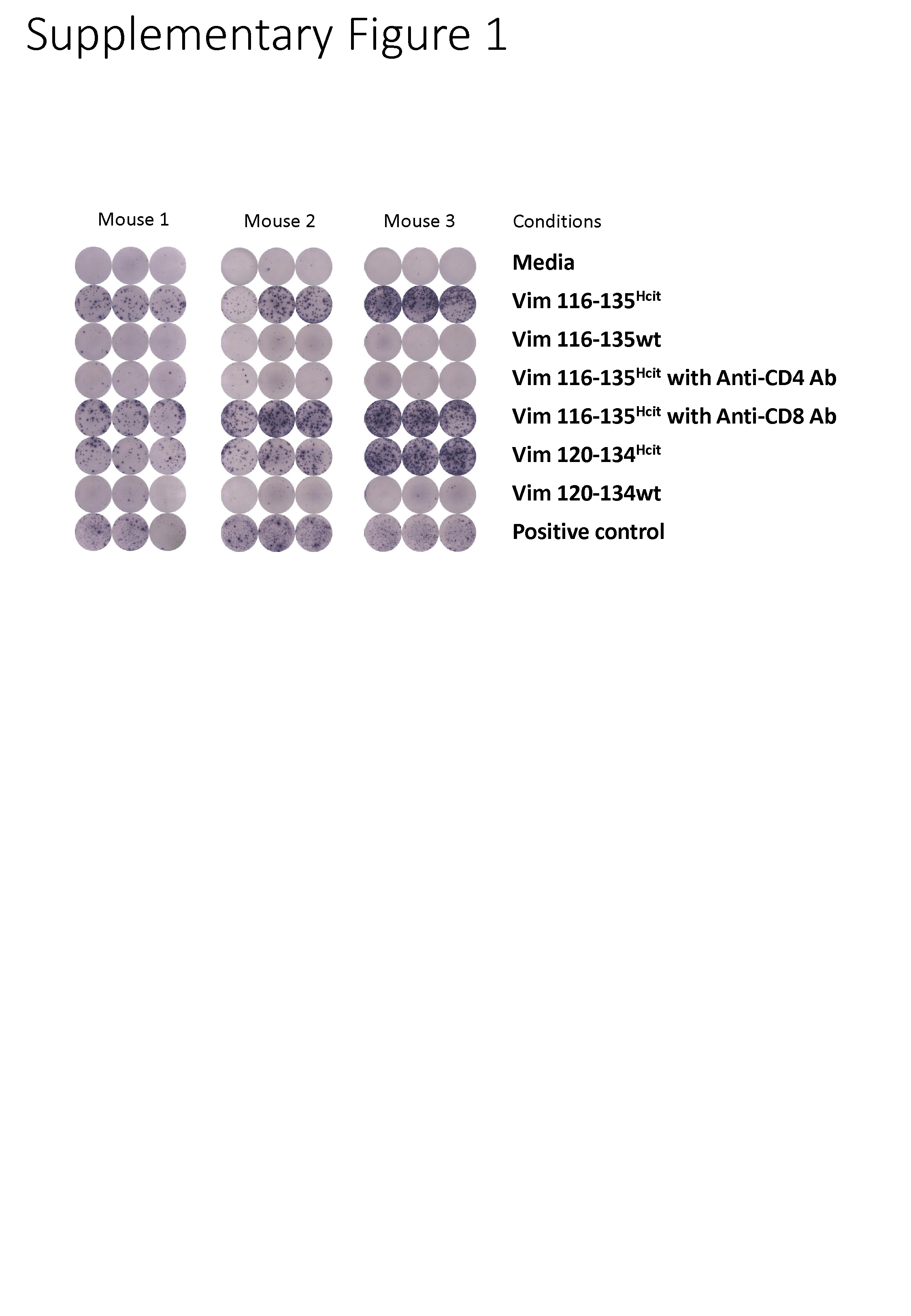

Supplement: Supplementary Figure 1 — Example ELISpot images. Example ELISpot images show HLA-HHDII/DR1 mice were immunized with the Vim116-135Hcit peptide. Responses for individual mice were assessed in triplicate to media only control, 10ug/ml peptides or positive control LPS. Responses to peptide were also assessed in the presence of anti-CD4 or anti-CD8 blocking antibodies. [file Image_1.tif]

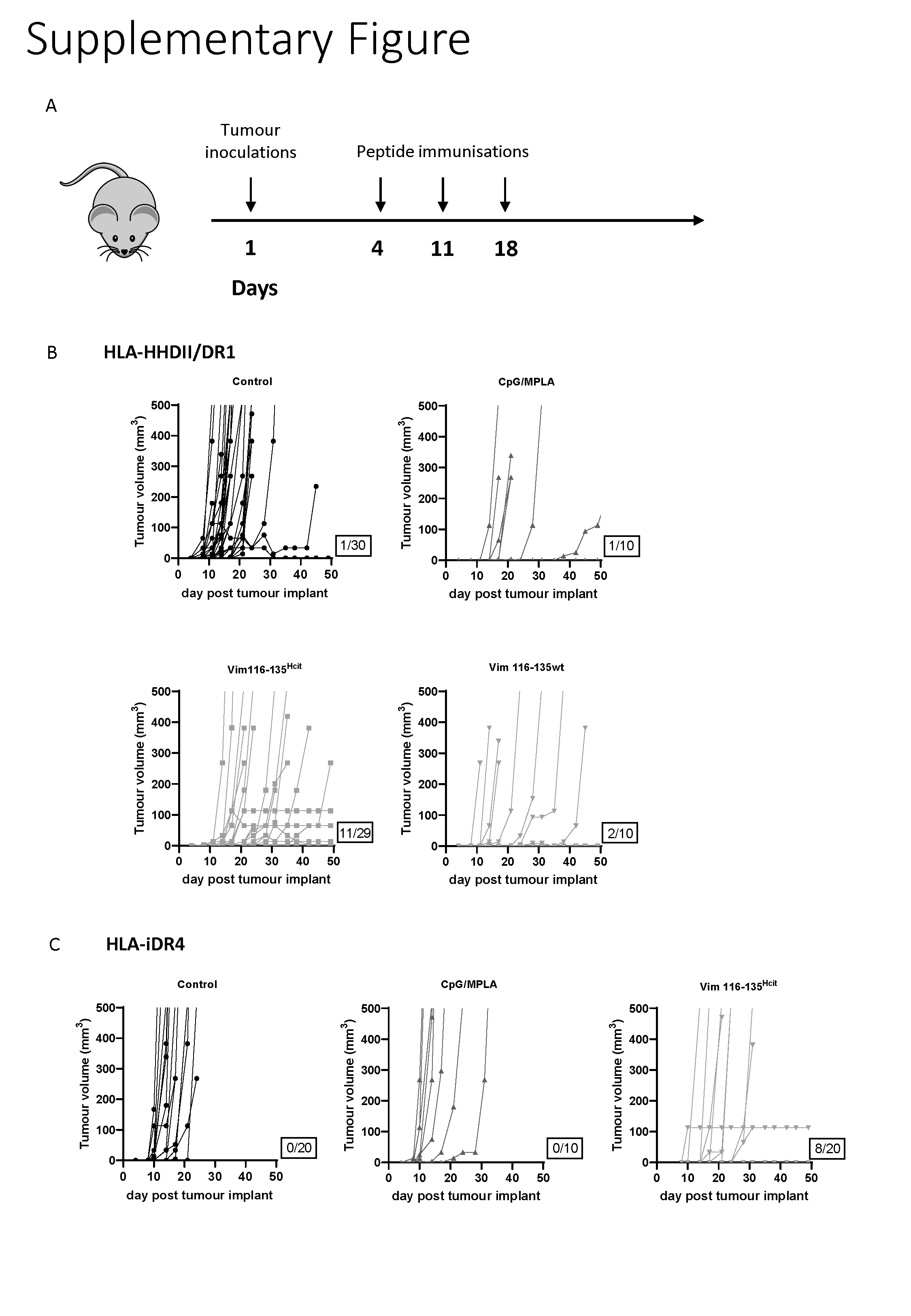

Supplement: Supplementary Figure 3 — Tumor growth curves. For all tumor studies mice were implanted with tumor on day 1 and then immunized on days 4, 11 and 18 (A). For some mice the home office license agreed humane end point was reached prior to final immunization and mice were terminated. HLA-HHDII/DR1 (B) and HLA-DR4 (C) mice were implanted with HLA-matched B16 F1 HHDII/DR1 (B) or HLA-iDR4 (C) cells. Mice were immunized with homocitrullinated (Hcit) or wild type (wt) Vim116-135 peptides or adjuvant only controls. Tumor growth for each group is shown. Boxed number represents the number of mice that were tumor-free at the end of the study for each group. [file Image_3.tif]
